# AI Technology Readiness, Trust, and Supervisor Academic Support in Relation to Graduate Students’ Research Self-Efficacy

**DOI:** 10.3390/bs16081313

**Published:** 2026-08-02

**Authors:** Yangyang Luo, Yingjie Fan, Tingting Wang, Qian Xu, Wanyi Zhao

**Affiliations:** 1 Institute of Higher Education, Lanzhou University, 222 Tianshui South Road, Chengguan District, Lanzhou 730000, China; luoyy@lzu.edu.cn (Y.L.); xu_qian@lzu.edu.cn (Q.X.); zhaowy2024@lzu.edu.cn (W.Z.); 2 School of Education, Renmin University of China, Haidian District, Beijing 100872, China; tingtingwang2024@ruc.edu.cn

**Keywords:** research self-efficacy, AI technology readiness, AI trust, supervisor academic support, PLS-SEM, graduate education

## Abstract

Research self-efficacy (RSE) is critical for graduate students. However, how emerging AI technologies shape RSE remains unclear. Grounded in the Technology Readiness Index (TRI) and Social Cognitive Theory, this study examined how AI technology readiness, AI trust, and supervisor academic support are associated with RSE. The study also examined whether these associations differ across disciplinary groupings. Cross-sectional data were collected from 425 master’s students at 19 Chinese universities. All participants self-reported regular use of AI in their research activities. The data were analyzed using partial least squares structural equation modeling (PLS-SEM) with multigroup analysis. Readiness facilitators were positively associated with AI trust and with perceived supervisor academic support. Readiness inhibitors were also positively associated with AI trust. Supervisor academic support was significantly associated with RSE. The direct association between readiness and RSE was not statistically significant. However, statistically significant indirect associations were observed via supervisor academic support and AI trust. Importantly, partial measurement invariance was established across groups. No between-group differences in path coefficients reached statistical significance. This indicates structural equivalence across the humanities and social sciences grouping and the science, engineering, and medicine grouping. The findings offer a preliminary extension of the TRI from adoption outcomes to efficacy beliefs in research training. They also inform tentative, discipline-uniform strategies for AI literacy education and supervisory support among master’s students who regularly use AI for research purposes.

## 1. Introduction

Self-efficacy represents a core construct in social cognitive theory, referring to individuals’ beliefs regarding their capabilities to successfully perform specific tasks, which subsequently influences their behavioral choices, effort expenditure, and persistence (Bandura, 1977). Research self-efficacy (RSE) extends this construct to the academic domain, referring to individuals’ beliefs and evaluative judgments regarding their capacity to successfully perform research-related tasks, including the formulation of research questions, study design, data analysis, and scholarly writing (J. Ren & Li, 2024). RSE demonstrates robust associations with academic interest, scholarly identity, intentions to pursue academic careers, research productivity, attitudes toward research, and working alliances with supervisors (Livinƫi et al., 2021). As central participants in graduate-level research activities, master’s students occupy a pivotal developmental stage characterized by the concurrent formation of research competence and scholarly identity (Jung, 2021). Their learning experiences during the master’s stage exert significant influence on future academic career choices and developmental trajectories (Li et al., 2024). The level of RSE among master’s students has been shown to predict both immediate academic outcomes—including learning engagement and academic performance (Miao et al., 2025)—and longer-term research productivity (Ndiango et al., 2024). Moreover, master’s education serves not only as a talent pipeline for doctoral programs, but also cultivates advanced professionals with specialized knowledge and research capabilities for diverse industries, constituting a vital component of national efforts to construct high-quality education systems. Therefore, investigation into factors influencing research self-efficacy among master’s students holds particular significance.

Social Cognitive Theory posits that individuals can enhance self-efficacy through four principal sources of information: mastery experiences, vicarious learning, verbal persuasion, and physiological and emotional arousal (Bandura, 1997). In traditional research training contexts, graduate students’ research self-efficacy has primarily stemmed from internal factors encompassing personal attributes and accumulated successful experiences, as well as external factors including family support, peer support, and supervisor support (Gong et al., 2022).

The rapid proliferation of artificial intelligence in higher education has, however, reshaped established academic practices. AI tools have progressively assumed the role of research aids, becoming increasingly embedded in graduate students’ learning and research workflows (Vinoth et al., 2025; Choudhari et al., 2025). Ofem et al. (2024) demonstrated that AI utilization significantly enhanced students’ perceived efficacy across multiple research phases, including background framing, literature synthesis, methodological application, and analytical reporting. Beyond task-level support, AI has also been recognized for its capacity to facilitate hypothesis generation, foster creative ideation, challenge conventional cognitive frameworks, deliver personalized learning resources, and scaffold collaborative academic discourse (Y. Liu & Xu, 2024).

For master’s students, the use of AI tools is associated with gains in learning efficiency and academic performance, but it also brings risks such as over-reliance, declines in critical thinking abilities, and ethical concerns (Hasin et al., 2025). Whether students can capture these benefits while avoiding the associated risks depends, in part, on their technology readiness, defined as individuals’ propensity to embrace and use new technologies (A. Parasuraman, 2000; A. Parasuraman & Colby, 2015). Bubou and Job (2022) noted that technology readiness is linked to whether students can appropriately select, comprehend, and utilize technology for learning, and a study on blended learning found that students’ technology readiness was positively associated with their learning motivation (Geng et al., 2019). Technology readiness can thus be regarded as a dispositional basis for engaging AI tools effectively in academic work. In the absence of adequate technology readiness, students are likely to encounter navigational difficulties with AI tools, experience impediments in AI-assisted research processes, and face heightened exposure to associated ethical risks. Meanwhile, trust in AI is equally crucial. Glikson and Woolley (2020) established that the effective integration of AI into collaborative settings is critically contingent upon individuals’ trust in AI systems. In educational contexts, insufficient trust in AI tools may substantially attenuate the anticipated benefits of AI adoption in higher education (F. Wang et al., 2025).

These constructs have, to date, been examined largely within separate research traditions. Within the technology-adoption literature, frameworks such as the Technology Acceptance Model (Davis, 1989), the Unified Theory of Acceptance and Use of Technology (Venkatesh et al., 2003), and the Technology Readiness Index (A. Parasuraman, 2000; A. Parasuraman & Colby, 2015) have predominantly explained behavioral intention and use behavior as outcomes; for example, Zhao et al. (2025) applied TRI 2.0 to examine how readiness facets drive or inhibit EFL learners’ translation technology use. Within the research-training literature, by contrast, models of research self-efficacy have emphasized supervisory and personal antecedents while giving limited attention to technology-related predispositions (J. Ren & Li, 2024; Kang et al., 2024). Two limitations of the existing literature follow. First, no prior model has jointly integrated technology readiness, AI trust, and supervisor support as antecedents of research self-efficacy; consequently, it remains unclear whether technology-related predispositions and human supervisory support are associated with research self-efficacy in complementary or competing ways in AI-mediated research practice. Although Huang et al. (2025) suggested that supervisor academic support and AI technical support may form a complementary alliance in facilitating positive research experiences, direct empirical evidence for this interplay remains scarce.

To address these gaps, the present study integrates the Technology Readiness Index framework with the research self-efficacy literature by examining how AI technology readiness, AI trust, and supervisor support are jointly associated with master’s students’ research self-efficacy, while incorporating disciplinary background as a moderating variable. The study is guided by two research questions: (1) How are AI technology readiness, AI trust, and supervisor support associated with master’s students’ research self-efficacy? and (2) Do these associations differ between the humanities/social sciences and science/engineering/medicine disciplinary groupings? Employing partial least squares structural equation modeling (PLS-SEM) with multigroup analysis, this study analyzes responses from 425 master’s students at multiple Chinese universities, thereby providing empirical evidence to inform the design of graduate cultivation strategies for higher education practitioners and policymakers in the AI era.

## 2. Research Model and Hypotheses Development

### 2.1. Theoretical Background

The study was framed by Bandura’s Social Cognitive Theory (SCT) as applied to human behavior (Bandura, 1986), its metatheoretical basis being that human behavior is regulated by the interaction of personal, behavioral, and environmental influences (Bandura, 1986). Self-efficacy, an important component in SCT, refers to the belief of an individual in the odds of successfully completing a particular action or dealing with a certain circumstance (Bandura, 1977; Schwarzer & Warner, 2013) and is here conceptualized as a result of both personal and environmental influences. Among AI-enabled learning environments, we consider AI technology readiness and AI trust as personal factors and supervisor academic support as a significant environmental factor. Readiness for AI technology is defined as a dimension of an informed decision for individuals to accept and use AI technology (Blut & Wang, 2020), indicating how individual attributes influence their interaction with AI tools. AI trust represents students’ perception about the dependability and capability of AI systems and thus is a cognitive element that affects technology use (McKnight et al., 2002). In contrast, supervisor academic support is an essential environmental factor in graduate studies to offer guidance and feedback in addition to addressing students’ academic concerns in their closest academic environment. SCT posits that these personal and environmental components are interdependent and concurrently influence student orientation to AI-enabled research processes, leading to the development of students’ beliefs about their ability to conduct research (i.e., their research self-efficacy).

### 2.2. AI Technology Readiness

Technology readiness is based on A. Parasuraman’s (2000) Technology Readiness Index (TRI) model and its extensions (A. Parasuraman, 2000). In this context, “optimism” and “innovativeness” are the facilitators and “insecurity” and “discomfort” the inhibitors (A. Parasuraman & Colby, 2015). In this context, facilitators are motivational forces exerting positive impacts on technology use acceptance, while inhibitors are motivational forces with negative impacts on use acceptance (Blut & Wang, 2020). As a trait-like individual difference, technology readiness captures individuals’ general predisposition to embrace new technologies (Blut & Wang, 2020). For the purpose of this research, AI technology readiness refers to an individual’s preparedness and inclination to employ and embrace AI applications in diverse educational activities. L. Ren et al. (2026) further found that AI has a significant positive impact on students’ self-efficacy in learning environments. These results serve as a basis for investigating the impact of technology readiness on research efficacy. Greater or higher levels of personal technology readiness lead to a higher perceived usefulness and ease of use of the technology, so that word-of-mouth adoption is more likely to occur (Blut et al., 2016). Particularly, the facilitators increase perceived competence in technology, positive judgments of its utilitarian benefit, and reduce perceptions of technical risk (Pham et al., 2020). Labrague et al. (2023) study shows individual technology readiness has a notable positive effect on technology self-efficacy. Research suggests that doctoral students who participate in iterative, high-interaction processes with AI-based assistive tools generally show superior writing performance (Nguyen et al., 2024). According to Social Cognitive Theory, facilitators positively influence graduate students’ intention to use AI in more specific research activities (topic selection, literature review, data analysis, and thesis writing) beyond the broad applications of academic writing and exam preparation. This increased readiness might predispose them to look for mastery through repeated practice, therefore improving their research self-efficacy. On the other hand, students who feel high levels of anxiety towards AI technology are more likely to experience frustration due to mismatches between affordances of technology and their academic needs. Those with a high level of insecurity tend to question the reliability of AI along with the risk of dependence, maintaining skeptical orientations towards AI-related research tasks—such orientations may dampen technology adoption intentions and in turn might reduce research self-efficacy (Hasan et al., 2024). In line with the above theoretical justification, the present study puts forward the following hypotheses. It is worth mentioning that these hypotheses are stated as general claims that are to hold over the disciplinary subsamples; any potential differences in magnitude or significance of these paths as a function of discipline should be treated as an empirical matter to be tested by means of multi-group analysis:

**H1a.**  *Facilitators of AI technology readiness positively and significantly relate to research self-efficacy.*

**H1b.**  *Inhibitors of AI technology readiness have a significant negative effect on research self-efficacy.*

### 2.3. Supervisor Academic Support

Academic support is considered to be the supervisor’s input into supporting the students’ academic and professional development through a number of means in order that they may accomplish their scholarly goals (Overall et al., 2011). In particular, supervisor academic support is operationalized as graduate students’ perceptions of the concrete academic task guidance provided by supervisors and the availability of support, as well as the timeliness of feedback. Based on Social Cognitive Theory, environmental support is a vital facilitating factor to translate one’s behavior potential into behavior performance (Bandura, 2001). Supervisors have been repeatedly shown to be the single most proximal environmental predictor of students’ research self-efficacy within the context of graduate education (Baker & Pifer, 2011). Supervisory input, including demonstrations of research methods and instruction in analyzing data, has been reported to enhance students’ belief in their own research skills. The literature has shown that supervisor academic support also positively impacts graduate students’ research self-efficacy (Kang et al., 2024).

Currently, the supervisor–student relationship has been largely impacted by the widespread use of artificial intelligence in higher education. AI can adapt learning content to students’ learning styles and speeds and help realize personalized learning, and it is becoming an increasingly important tool for students to obtain knowledge (Ma et al., 2024). AI technology has transformed access to disciplinary knowledge for students, but it also challenges and reshapes supervisory roles in graduate education. J. Liu and Peng (2025) pointed out that in the AI era, the role of supervisors themselves is evolving from “knowledge transmitters” to “learning facilitators.” Research on the effect of AI chatbots on teacher–student relations in HE showed that AI chatbots can facilitate productive interaction between supervisors and students as well as increase teaching efficiency (Zou, 2024). Importantly, Hong and Litwin (2025) also revealed that students’ expectations toward traditional thesis supervision have shifted as a result of the usage of AI: given that AI can answer low-level questions, students now expect to be guided on matters of content and the research process. Confirming that, and more, Huang et al. (2025) demonstrated that students utilizing AI technology in research are able to benefit from a more in-depth and higher-quality supervisor academic support. Therefore, it can be inferred that the same high levels of AI technology readiness among graduate students might be expected to both improve the efficiency of supervisor–student interactions, redirect supervisory content towards higher-order conceptual and methodological concerns, and ultimately increase students’ levels of perceived quality of academic support. In contrast, technology readiness inhibitors have been linked to increased AI anxiety (Lemay et al., 2020), which might restrict students’ appropriation of AI tools for core research activities, potentially negatively impacting the substantive nature of supervisor–student academic collaborations. Based on the above theoretical foundation, the following hypotheses for this study are proposed:

**H2a.**  *Facilitators of AI technology readiness have a significant positive effect on the academic support from the supervisors.*

**H2b.**  *AI technology readiness inhibitors affect supervisor academic support negatively.*

**H3.**  *Supervisor academic support significantly positively influences research self-efficacy.*

### 2.4. AI Trust

AI trust is defined as individuals’ basic perceptions of reliability, security, predictability and ethicality associated with AI technologies and systems within specific tasks (Jonelid et al., 2024). Based on McKnight et al.’s (1998) and McKnight et al.’s (2002) conceptualization of trust, this study defines AI trust as two distinct but interrelated dimensions: (a) trusting beliefs, which refers to the belief about the competence of AI to produce the desired academic outcomes, the integrity of the information generated by AI and the safety of its use, and (b) institution-based trust, which includes the belief that the adoption of AI in the graduate education system is situationally normal and is supported by governmental policy frameworks that help it. While previous studies have not specifically explored the influence of AI trust on research self-efficacy, Amoozadeh et al. (2024) found indirect evidence for the positive impact of AI trust on students’ learning performance; that is, AI trust positively correlated with learning motivation and task confidence in programming. In the university setting, the frequency of use, length of usage, and perceived competence (Đerić et al., 2025) all played a critical part in predicting AI trust. The facilitators of AI technology readiness are associated with a higher intention to accept and use AI-related technologies, with a strong positive influence on perceived usefulness and perceived ease of use (Blut et al., 2016). Subsequently, the trust in AI of the users is influenced by the perceived ease of use and perceived usefulness of the AI technology (Kurniawan et al., 2022). With the gradual solidification of AI trust based on repeated cognitive evaluation of AI means, students are able to find higher potential in conducting AI-assisted research tasks. This process aligns with SCT. It is postulated to produce cumulative mastery experiences, and such experience further bolsters their research self-efficacy.

Significantly, the inhibitory aspects encompass a number of issues. These comprise fears associated with inaccurate outputs, overreliance, privacy, and plagiarism. These may be concerns beyond resistance to technology, but rather more general concerns. Empirical investigations in the context of education support this interpretation. Students often raise concerns about misinformation, dependence, privacy, and academic honesty. They voice these worries in the very process of using generative AI (Chan & Hu, 2023). These are being described as expressions of ethical sensitivity and critical appraisal. They are not being framed as technophobia (Higgs & Stornaiuolo, 2024). Amongst daily AI multi-users, ingrained concerns will hence more likely be expressed in terms of critical reflections “while resources are consumed”. They are less prone to appear as an avoidance of use. The theory of trust calibration also posits that justified trust is based on realism. This evaluation includes the capabilities as well as the limitations of the system. It is not based on blind faith (R. Parasuraman & Riley, 1997; De Visser et al., 2020). Certain students are more sensitive to the fallibility of AI. These students may be more critical when evaluating AI outputs. They may also hold more correct mental representations of what AI can and cannot do when applied to research tasks.

As a mediating factor, trust in AI has been found to alleviate anxiety related to technology and promote continuous use of technology (Zhou et al., 2025). The mediating effect of AI trust is not limited to the decrease in anxiety; technology self-efficacy can also affect technology adoption through trust as a mediator (Montag et al., 2023). Drawing on the theoretical grounding described above, the following hypotheses are proposed with respect to this study:

**H4a.**  *The facilitators of AI technology readiness have a positive effect on AI trust.*

**H4b.**  *The inhibitors of AI technology readiness have a positive effect on AI trust.*

**H5.**  *AI trust has a significant positive effect on research self-efficacy.*

**Figure 1:**
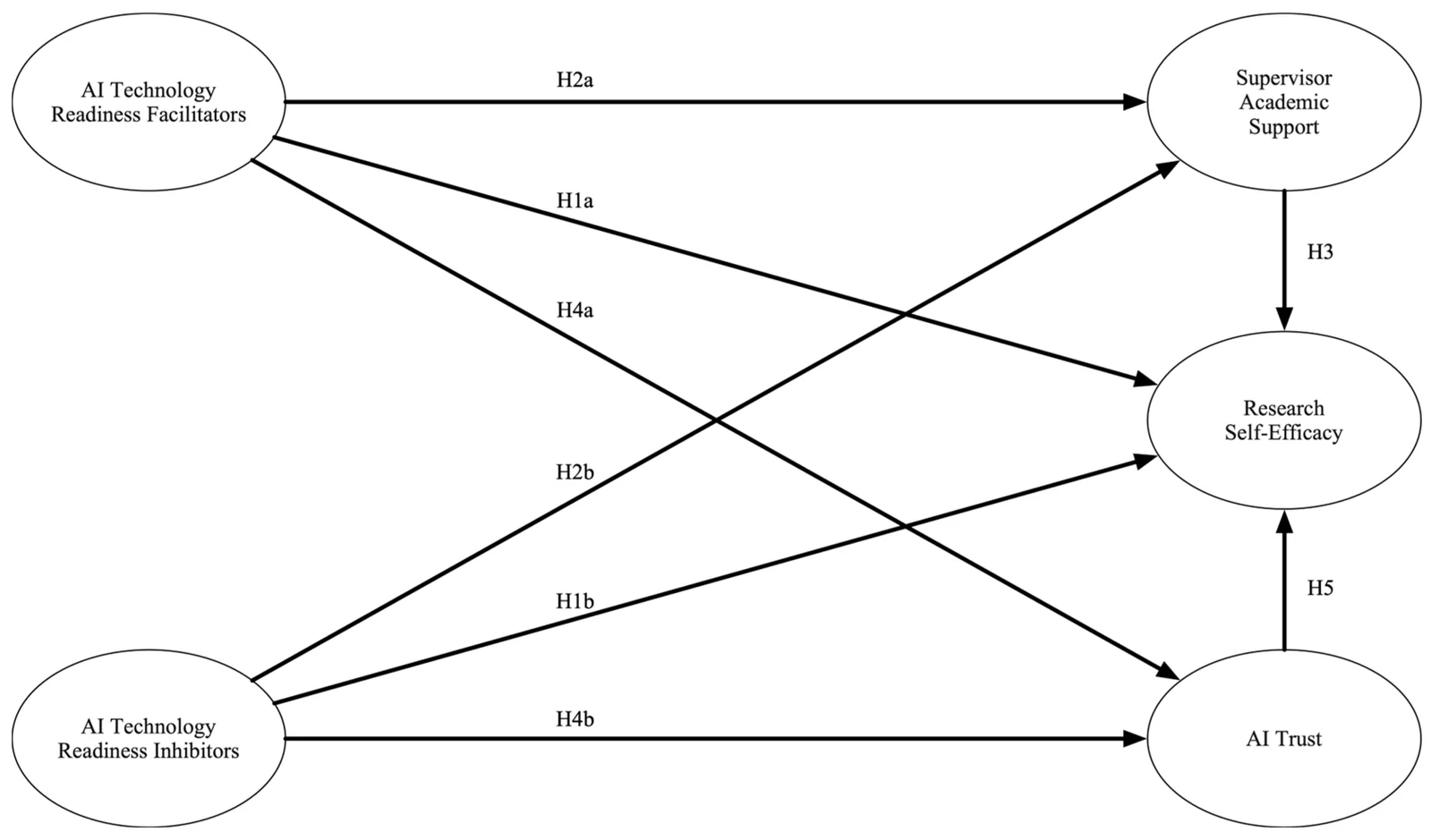
Proposed theoretical model.

In brief, the focus of this study is to test a model, as shown in Figure 1, hypothesizing the relationships among graduate students’ AI technology readiness, AI trust, supervisor academic support, and research self-efficacy:

## 3. Research Methodology

### 3.1. Questionnaire Design

This study used a cross-sectional design. All variables were collected through self-report questionnaires. To promote measurement rigor, the survey instrument was based on a set of well-established, psychometrically sound scales identified in the existing literature. All items apart from the demographic questions were rated on a 7-point Likert scale. As the original scales were in English, a back-translation procedure was followed according to recommended guidelines (Brislin, 1970; Beaton et al., 2000). Two subject-matter experts who are bilingual independently conducted the forward translation and reconciled discrepancies; three native Chinese-speaking graduate students then back-translated the items into English. An expert panel reviewed the back-translated version and the original version item by item for each of four criteria (Beaton et al., 2000): semantic, idiomatic, experiential, and conceptual equivalence and re-translated any item found not to be equivalent until the team reached consensus. A pilot study was conducted with a sample of 105 graduate students; data from this stage were not included in the main analyses. The Cronbach’s alphas for all the Constructs are above the recommended level of 0.70, indicating acceptable internal consistency and further scale revision was not required.

#### 3.1.1. AI Technology Readiness Scale

The scale of AI readiness in technology included 16 items, which were mainly derived from the instrument by Sudaryanto et al. (2023) to measure the readiness of graduate students to use AI technology (Table A1). This scale has four factors: the first four questions, 1 to 4, assess the optimism dimension; questions 5 to 8 assess the innovativeness dimension; questions 9 to 12 assess the discomfort dimension; and questions 13 to 16 assess the insecurity dimension. Optimism represents graduate students’ positive expectations and beliefs about the potential of AI technology to improve learning, research, and personal productivity. This dimension essentially represents a predisposition to expect positive outcomes of using AI, such as greater convenience, efficiency, and ability to solve problems. One example of a question is “AI technology enables me to better manage learning and research tasks in daily life in mobile scenarios.” Innovativeness is a graduate student’s innate motivation and tendency to actively acquire, research, and use tools of AI technology in their professional areas. This factor represents the extent to which a person is open to new technology, has the desire to learn, and has the confidence to learn and use technology on their own (A. Parasuraman & Colby, 2015). A sample item is “I am able to independently seek out and learn relevant AI technology tools that are related to my major.” “Discomfort” is a subjective experience in which graduate students feel frustrated and/or maladapted, encountering a tension or discrepancy between the technology and their own needs or usage habits while they use AI during its utilization. This discomfort can be caused by technology design, by fundamental technical restraints, or by the mismatch between expectation and performance. A typical item includes: “The development of some AI tools seems not to have taken adequately into account the real needs of typical graduate student users.” Insecurity refers to graduate students’ perceptions of the security risks of AI systems, technological risks, and negative implications of reliance on AI-based outputs. An example item includes: “Excessive trust in AI technology may hinder my concentration and even guide me to incorrect content.”

#### 3.1.2. Supervisor Academic Support Scale

The Supervisor Academic Support Scale was adapted from the instrument developed by Overall et al. (2011), comprising 14 items (Table A2). This scale consists of two factors. The first factor assesses support related to academic tasks, including items such as “My supervisor establishes clear research goals for me to achieve” and “My supervisor seeks out information helpful for my thesis/research.” The second factor evaluates the availability of supervisor support and the timeliness of feedback provision, including items such as “My supervisor responds to my questions or requests within a reasonable time frame.”

#### 3.1.3. Research Self-Efficacy Scale

The Research Self-Efficacy Scale comprises 15 items adapted from the instrument developed by Pentang and Domingo (2024) (Table A3). Although graduate students have yet to become formal researchers, they constitute pre-service researchers; therefore, this scale is applicable to master’s student populations. Research self-efficacy reflects researchers’ capability beliefs when completing various research tasks, emphasizing researchers’ self-evaluations of their abilities to accomplish research tasks and their confidence in mastering research methodologies. Sample items include: “I am capable of systematically collecting and organizing research data (e.g., experimental operations, questionnaire surveys, transcription of in-depth interviews, or text coding)” and “I am proficient in retrieving literature on specific research topics using Chinese and English literature databases (e.g., CNKI, Web of Science).”

#### 3.1.4. AI Trust Scale

The AI Trust Scale comprises 5 items adapted from the instrument developed by Shahzad et al. (2025) (Table A4), primarily assessing graduate students’ level of trust in using AI for learning. Sample items include: “I believe that using artificial intelligence can help me achieve better grades in my studies” and “I believe that using artificial intelligence in my learning is safe and reliable.”

### 3.2. Data Collection

The data for this study were collected from Wenjuanxing, which is a professional online survey platform and is widely used in Chinese academic research (P. Wang et al., 2026; Yang et al., 2026). Convenience sampling was used. The questionnaire link was shared with master’s students of 19 Chinese universities via graduate student online communities, WeChat groups, and emails through faculty contacts at each institution. All participation was fully voluntary; respondents were notified of the purpose of the research and had given consent for the study prior to answering the questionnaire. There was complete anonymity and confidentiality for the responses, and all the collected data were only used for academic purposes in line with the standard of research ethics. To meet the goals of the study and obtain truthful responses, the following two inclusion criteria were implemented: (1) a single-choice screening question asking whether they used artificial intelligence regularly in their research must be answered with a “Yes”; and (2) they were currently pursuing a master’s degree. Therefore, this study’s target population can be considered as those Chinese master’s students who conduct research using AI regularly, as opposed to all master’s students. In a six-week period of data collecting, 463 questionnaires were received overall. After removing 27 responses with suspiciously short response times and 11 respondents who did not satisfy the AI-use criterion, 425 valid responses were left for analysis, which corresponded to an effective response rate of 91.8%. Table 1 displays the final sample, which consisted of 425 master’s students from different Chinese universities. A total of 280 respondents (65.9%) were female, and 145 (34.1%) were male. Among them, 215 respondents (50.6%) were from urban areas while 210 (49.4%) were from rural areas in terms of native place. With regard to disciplinary fields, it consisted of engineering *n* = 158 (37.2%), science *n* = 65 (15.3%), education *n* = 61 (14.4%), law *n* = 43 (10.1%), management *n* = 25 (5.9%), medicine *n* = 21 (4.9%), arts *n* = 18 (4.2%), literature *n* = 15 (3.5%), economics *n* = 11 (2.6%), history *n* = 4 (0.9%) and philosophy *n* = 4 (0.9%). All were enrolled master’s students who indicated that they regularly used AI in their research work, which matches the inclusion criteria at the beginning.

### 3.3. Data Analysis Strategy

Structural Equation Modeling (SEM) integrates factor analysis and path analysis. It estimates complex multivariate relationships among latent variables. In this study, partial least squares SEM (PLS-SEM) was selected over covariance-based SEM (CB-SEM) for three reasons. First, this study has an exploratory character. It extends the Technology Readiness Index to a new outcome, research self-efficacy, in the context of AI-assisted graduate research. These relationships have not been tested in this context before. PLS-SEM is designed for exploratory research and for the extension of existing theory. It is not intended for strict confirmation of a well-established model (J. F. Hair et al., 2019, 2022). Second, the data violate multivariate normality assumptions. PLS-SEM does not require normally distributed data. Third, the research model is relatively complex. It comprises five latent constructs and 48 measurement indicators. It also includes two indirect pathways, via AI trust and via supervisor academic support.

The PLS-SEM algorithm and bootstrapping were performed using SmartPLS 4.0 (Version 4.1.0.6) to evaluate the measurement model and structural model. First, the measurement model was assessed to ensure the reliability and validity of the hypothesized model. The measurement model was evaluated using the following criteria: outer loadings, Cronbach’s alpha, composite reliability (CR), average variance extracted (AVE), and the heterotrait–monotrait (HTMT) ratio of correlations among all variables in the model (J. Hair & Alamer, 2022). Second, the structural model was evaluated. Bootstrapping was employed to examine the significance and strength of the hypothesized paths between latent variables. In addition, the effect size *f*^2^ was assessed for each direct path to evaluate the substantive relevance of the structural relationships. Finally, to examine whether the structural relationships differed across disciplinary groups, a multigroup analysis (MGA) was conducted, with respondents divided into a humanities and social sciences group and a science, engineering, and medicine group.

## 4. Results

### 4.1. Common Method Bias

To statistically assess the presence of CMB, a confirmatory factor analytic (CFA) version of the single-factor test was employed in preference to the traditional exploratory Harman’s single-factor test. The traditional approach, which merely examines the proportion of variance accounted for by the first unrotated factor in an exploratory factor analysis, has been widely criticized for its limited sensitivity in detecting method variance and its inability to provide a formal statistical test of whether a single factor adequately accounts for the covariation among items (Podsakoff et al., 2003, 2024). By contrast, the CFA-based approach constrains all measurement items to load on a single latent factor, thereby permitting a formal evaluation of model fit as well as a nested chi-square difference test against the hypothesized multi-factor measurement model. Accordingly, all 48 measurement items were constrained to load on one factor, and the analysis was conducted on the full sample. The resulting one-factor model demonstrated poor fit to the data (χ^2^/df = 16.385; GFI = 0.169; CFI = 0.471; TLI = 0.450; RMSEA = 0.190; SRMR = 0.207), and a chi-square difference test confirmed that it fitted the data significantly worse than the hypothesized multi-factor measurement model (Δχ^2^ = 12,724.82, Δdf = 10, *p* < 0.001). These results indicate that a single method factor cannot adequately account for the covariation among the items, suggesting that common method bias is unlikely to pose a serious threat to the validity of the study’s findings (Podsakoff et al., 2003).

### 4.2. Reliability and Validity of the Measurement Model

Prior to hypothesis testing, the measurement model was assessed. The evaluation results indicated that the data fit was acceptable. In accordance with established criteria (J. Hair & Alamer, 2022), standardized outer loadings were required to meet or exceed the threshold of 0.700, with both Cronbach’s alpha (α) and composite reliability (CR) similarly required to surpass 0.700 (J. Hair & Alamer, 2022). During measurement model evaluation, items RSE14 and RSE15 were excluded on the grounds that their standardized outer loadings fell below the prescribed threshold of 0.700. Notably, both removed items assessed compliance with academic ethical norms and integrity rules; their low loadings suggest that confidence in ethical compliance—behavior governed by normative rules rather than perceived task capability—may be conceptually distinct from the task-oriented research self-efficacy captured by the retained items (Bandura, 1997). As shown in Table A5, after removing RSE14 and RSE15, the outer loadings for all items across variables ranged from 0.742 to 0.964, meeting the criteria for individual item reliability.

Regarding validity assessment, Average Variance Extracted (AVE) values should exceed 0.500 (J. Hair & Alamer, 2022). In the present study, AVE values for all variables exceeded the critical threshold of 0.50, indicating adequate convergent validity. For discriminant validity assessment, J. Hair and Alamer (2022) recommended employing the Heterotrait–Monotrait (HTMT) ratio of correlations. Regarding threshold criteria, the HTMT confidence intervals should not include the value of 1, preferably falling below the critical value of 0.90, or more conservatively, 0.85 (Henseler et al., 2015). As presented in Table A6, HTMT values for all variables ranged from 0.340 to 0.792, with each value falling below the conservative threshold of 0.85. Taken together, the preceding results provide empirical support for the discriminant validity of the measurement model.

### 4.3. Structural Model Assessment

Structural model assessment typically involves examining the *R*^2^ values of endogenous variables and evaluating the significance and relevance of path coefficients (J. Hair & Alamer, 2022). This study conducted structural model testing using SmartPLS 4.0 software (Version 4.1.0.6). *R*^2^ represents the proportion of variance in the dependent variables explained by the model, where *R*^2^ values of 0.19, 0.33, and 0.67 indicate weak, moderate, and substantial explanatory power, respectively (Chin, 1998). The test results are presented in Table 2. The *R*^2^ values were 0.257 for supervisor academic support, 0.586 for AI trust, and 0.486 for research self-efficacy. These results indicate that the endogenous latent variables in models were adequately explained.

Furthermore, this study assessed the *Q*^2^ values of the structural models. *Q*^2^ serves as an indicator of predictive relevance (Chin, 1998). In this study, *Q*^2^ values were 0.242 for supervisor academic support, 0.581 for AI trust, and 0.294 for research self-efficacy. All *Q*^2^ values exceeded zero, indicating that the structural model exhibited adequate predictive relevance.

To ensure model robustness, Variance Inflation Factor (VIF) values for predictor variables are preferably kept below 3, though a more lenient threshold of below 5 is also acceptable to ensure that multicollinearity does not exert substantial influence on structural model estimates (J. Hair & Alamer, 2022). As shown in Table 3, all VIF values remained below 3. Collectively, VIF values for model fell within acceptable ranges, indicating that indicators demonstrated high contributions to their respective constructs without multicollinearity concerns.

### 4.4. Path Analysis and Hypothesis Testing

Following the recommendations of J. F. Hair et al. (2022), this study employed bootstrapping with 10,000 subsamples, two-tailed tests, and a significance level of 0.05 to obtain standard errors and *t*-statistics. In addition, the effect size *f*^2^ was assessed to evaluate the substantive relevance of the direct paths; following Cohen (1988), *f*^2^ values of 0.02, 0.15, and 0.35 were interpreted as small, medium, and large effects, respectively. Table 4 presents the results of the research model.

AI technology readiness facilitators showed significant positive associations with supervisor academic support (β = 0.460, *t* = 6.029, *p* < 0.001) and AI trust (β = 0.665, *t* = 15.365, *p* < 0.001), but not with research self-efficacy (β = 0.117, *t* = 1.415, *p* = 0.157). Thus, Hypotheses H2a and H4a were supported, whereas Hypothesis H1a was not supported. AI technology readiness inhibitors showed a significant positive association with AI trust (β = 0.157, *t* = 3.486, *p* < 0.001), but no significant associations with supervisor academic support (β = 0.075, *t* = 1.072, *p* = 0.284) or research self-efficacy (β = 0.019, *t* = 0.365, *p* = 0.715). Consequently, Hypothesis H4b was supported, while Hypotheses H2b and H1b were not supported. Supervisor academic support was positively associated with research self-efficacy (β = 0.436, *t* = 6.016, *p* < 0.001), supporting Hypothesis H3. AI trust was also positively associated with research self-efficacy (β = 0.266, *t* = 3.781, *p* < 0.001); thus, Hypothesis H5 was supported.

Regarding indirect effects, the results indicated statistically significant indirect effects of AI technology readiness facilitators on research self-efficacy via supervisor academic support (β = 0.201, *t* = 4.478, *p* < 0.001) and via AI trust (β = 0.177, *t* = 3.695, *p* < 0.001). For AI technology readiness inhibitors, the indirect effect on research self-efficacy via AI trust was statistically significant (β = 0.042, *t* = 2.493, *p* = 0.013), whereas the indirect effect via supervisor academic support was not significant (β = 0.033, *t* = 1.036, *p* = 0.300), as its confidence interval included zero. Considered alongside the non-significant direct effects, the total indirect effects of facilitators (β = 0.378, *t* = 6.165, *p* < 0.001) and inhibitors (β = 0.075, *t* = 2.197, *p* = 0.028) indicate that research self-efficacy is associated with AI technology readiness primarily through statistically significant indirect pathways rather than direct ones.

### 4.5. Multigroup Analysis

To examine whether the structural relationships in the research model differed across disciplinary contexts, a multigroup analysis (MGA) was performed. Consistent with the discipline-based stratification of the sample, respondents were divided into two groups: humanities and social sciences (*n* = 181) and science, engineering, and medicine (*n* = 244). Regarding sample classification, this study followed the Graduate Education Discipline Catalogue (2022) issued by the Academic Degrees Committee of the State Council and the Ministry of Education of China. The humanities and social sciences group included Literature, History, Arts, Philosophy, Economics, Law, Education, and Management. The aggregation of the first-level disciplines into two broad groupings was based on both theoretical and methodological considerations. Theoretically, this dichotomy corresponds to the well-established “soft–hard” dimension of disciplinary classification (Biglan, 1973; Becher & Trowler, 2008). Within this framework, humanities and social sciences disciplines are characterized by interpretive, text-centered research practices, relatively individualized supervisory models, and constructivist epistemological traditions, whereas science, engineering, and medicine disciplines are characterized by experimental, data- and instrument-centered research practices, team- or laboratory-based supervisory models, and positivist epistemological traditions. Prior to comparing the groups, measurement invariance was assessed using the measurement invariance of composite models (MICOM) procedure (Henseler et al., 2015), which involves configural invariance (Step 1), compositional invariance (Step 2), and the equality of composite means and variances (Step 3).

Configural invariance was established by employing identical indicators, data treatment procedures, and algorithm settings for both groups. As shown in Table 5, compositional invariance was confirmed for all constructs, with the original correlations equal to or exceeding the 5.0% permutation quantile, thereby establishing partial measurement invariance. Although full measurement invariance was not supported for two constructs (TRIB and S) due to unequal composite variances, partial measurement invariance is considered a sufficient condition for meaningful multigroup comparisons (Henseler et al., 2015; J. F. Hair et al., 2022). Between-group differences in path coefficients were then assessed using the permutation test with 5000 permutations.

The multigroup analysis (Table 6) compares the path coefficients between the science/engineering/medicine (SEM) and humanities/social sciences (HSS) samples. The association between supervisor academic support and research self-efficacy was statistically significant in both the SEM (β = 0.463, *p* < 0.001) and HSS (β = 0.383, *p* < 0.001) samples, and the difference between the coefficients was not statistically significant (Henseler’s MGA *p* = 0.525; permutation *p* = 0.612). Similarly, the paths from AI technology readiness facilitators to AI trust (SEM: β = 0.722, *p* < 0.001; HSS: β = 0.550, *p* < 0.001), from facilitators to supervisor academic support (SEM: β = 0.395, *p* < 0.001; HSS: β = 0.601, *p* < 0.001), and from inhibitors to AI trust (SEM: β = 0.122, *p* = 0.028; HSS: β = 0.237, *p* = 0.004) were statistically significant in both groups, with no significant between-group differences (all *p* > 0.05). The association between AI trust and research self-efficacy was statistically significant in the SEM sample (β = 0.352, *p* = 0.001) but not in the HSS sample (β = 0.167, *p* = 0.075); nevertheless, the difference between the coefficients was not statistically significant (Henseler’s MGA *p* = 0.185; permutation *p* = 0.193). Likewise, the direct association between facilitators and research self-efficacy was significant in the HSS sample (β = 0.241, *p* = 0.020) but not in the SEM sample (β = 0.014, *p* = 0.901), while the between-group difference again did not reach statistical significance (Henseler’s MGA *p* = 0.145; permutation *p* = 0.186). The paths from inhibitors to supervisor academic support and to research self-efficacy were not significant in either group, nor did they differ significantly between groups.

Regarding indirect effects, the indirect association of AI technology readiness facilitators with research self-efficacy via supervisor academic support was statistically significant in both samples (SEM: β = 0.183, *p* = 0.002; HSS: β = 0.230, *p* < 0.001), whereas the indirect association via AI trust was significant in the SEM sample (β = 0.254, *p* = 0.001) but not in the HSS sample (β = 0.092, *p* = 0.085). In the SEM sample, the indirect association of AI technology readiness inhibitors with research self-efficacy via AI trust was small but statistically significant, whereas the corresponding indirect association was not significant in the HSS sample; the indirect associations of inhibitors via supervisor academic support were not statistically significant in either group. However, none of the between-group differences in the indirect effects were statistically significant (all Henseler’s MGA and permutation *p*-values > 0.05). Statistical significance of all within-group effects was determined on the basis of bias-corrected and accelerated (BCa) bootstrap confidence intervals (J. F. Hair et al., 2022). It should be noted that differences in within-group significance do not imply significant between-group differences, which were assessed directly using Henseler’s MGA and the permutation test. Overall, the structural relationships in the research model were statistically equivalent across the two disciplinary groups.

Overall, the results indicate that disciplinary background does not significantly moderate the proposed relationships. This suggests that the mechanisms linking AI technology readiness, AI trust, supervisor academic support, and research self-efficacy operate uniformly across disciplinary contexts. This finding underscores the structural invariance of the proposed model. It also highlights the generalizability of the model among graduate students from different disciplinary groups. The two groups exhibited distinct within-group significance patterns. Facilitators directly predicted research self-efficacy only in the humanities and social sciences group. AI trust was a significant predictor only in the science, engineering, and medicine group. However, these patterns are interpreted as exploratory rather than confirmatory. This interpretation is based on the observation that none of the corresponding coefficient differences reached statistical significance.

## 5. Discussion

### 5.1. Relationship Between Artificial Intelligence Technology Readiness and Trust in AI

The results indicate that the facilitators of AI technology readiness had positive and significant relationships with trust in AI. The result supports the application of the Technology Readiness Index (TRI) (A. Parasuraman & Colby, 2015) across disciplines in higher education. The technology readiness facilitators have a strong indirect effect on technology adoption via perceived usefulness and perceived ease of use, as indicated in the meta-analysis of Blut and Wang (2020). More specifically, facilitators facilitate the development of trust by increasing individuals’ positive expectations about the effectiveness of AI tools as well as convenience of use (Kurniawan et al., 2022). Technology readiness is a key antecedent for predicting graduate students’ trust in AI in the context of higher education. AI technology readiness inhibitors were also found to be positively and significantly related to AI trust. This is the opposite of the prevailing belief that inhibitors slow down the acceptance of technology (A. Parasuraman & Colby, 2015). A possible reading is that these inhibitor-related anxieties are those of critical scrutiny rather than technological hesitance. Such a reading aligns with recent work on critical thinking and AI use. Such work frames critical thinking as fact-checking outputs generated by AI, an interest in understanding the limits of AI, and a consideration of responsible application. This disposition is associated with more accurate assessments of AI-produced information (Lau et al., 2026). Hoff and Bashir (2015) made a distinction between dispositional and learned trust. They showed that early technology anxiety could be changed into strong, experience-based trust through iterative assessment of experiences. In the present context, graduate students with AI insecurity or anxiety may be inclined to adopt more cautious, reflective, and proactive learning strategies when using AI applications. In turn, this process may allow experiential justification to emerge sufficient to allow for rational trust formation. Such description is consistent with Rathakrishnan et al.’s (2024) finding that early AI anxiety may facilitate prudent cognitive appraisal and help-seeking behaviors, leading to positive acceptance intentions. In addition, as decision stakes rise, people appear to adopt even stricter standards of evaluation in advance of granting their trust to AI systems (Horowitz & Kahn, 2024). This seems like it might paradoxically cultivate rather than erode the trust that is ultimately built.

### 5.2. The Relationship of AI Technology Readiness and Supervisor Academic Support

The results showed that facilitators of AI technology readiness were positively and significantly related to supervisor academic support. Although previous studies have not specifically explored the connection between personal technology readiness and perceived social support in higher education, the current results align with those of Huang et al. (2025), who argued that students’ use of AI tools in research activities leads to increased depth and quality of supervisor academic support. This pattern may be due to the ability of AI to provide real-time, individualized feedback from tutors, which shortens the distance between what learners are currently capable of doing and what they should learn to do (Mollick & Mollick, 2023). Cope and Kalantzis (2017) findings suggest that AI has the potential to reduce time spent on literature review significantly for supervisors and students, enabling them to focus on more advanced conceptual and methodological issues.

Interestingly, none of the AI technology readiness inhibitors were significantly related to supervisor academic support. Based on Social Cognitive Theory’s concept of human agency (Bandura, 2001), people can actively and proactively modify their thoughts, moods, and behavioral orientations. The positive attitudes associated with readiness facilitators may lead students to be more active in exploring technology, which could also enhance the quality of the substantive content in the interactions between supervisors and students. Such interaction results may then alter students’ cognitive appraisals of the quality of supervisory support and thus their perceived level of academic support. Meanwhile, none of the inhibitors showed a direct path to perceptions of supervisor support. This result converges with the Social Cognitive Theory core principle that behavior is the primary interconnecting the person and environmental factors (Bandura, 2001) and indicates that negative technology-related cognitions do not stunt perception of external social support.

### 5.3. The Association Between AI Technology Readiness and Research Self-Efficacy

Supervisor academic support was positively related to research self-efficacy. This is consistent with J. Ren and Li (2024) finding that academic guidance, suggestions on research methodology, timely feedback, and directive input from supervisors have the potential to directly foster graduate students’ research self-efficacy. In addition to the direct link, AI technology readiness facilitators were significantly and positively indirectly related to research self-efficacy through supervisor academic support. The direct relation between facilitators and research self-efficacy was not significant. It implies that this link operates via a relational route, primarily. They hold more positive attitudes towards AI-assisted research. This positive consideration may also encourage more substantive supervisor-student dialogues. Such exchanges may revolve around higher-level conceptual and methodological concerns (Cope & Kalantzis, 2017; Mollick & Mollick, 2023). This interpretation is consistent with Social Cognitive Theory. Bandura (1997) stressed that self-efficacy is partly based on environmental and social sources of information. These factors encompass supervisory guidance and verbal persuasion. This is consistent with organisation-level data. Park et al. (2018) indicated that supervisor support connects individual capability inputs with outcome performance.

Research self-efficacy was not significantly affected by inhibitors of AI technology readiness. To our knowledge, there has been no research investigating the effect of individual technology readiness on self-efficacy in higher education environments. This can be explained by SCT, which claims that people increase their self-efficacy through mastery experiences, vicarious learning, verbal persuasion and physiological and emotional arousal (Bandura, 1997). While inhibitors of AI technology readiness represent the graduate students’ psychological unease and uncertainty about AI, such dispositions undoubtedly do not prevent students from engaging in research pursuits by traditional means. This implies that AI technology has yet to become a compulsory “must-know” for graduate research outputs in China, and such inhibitory predispositions may not be that influential as to be construed as detriments to students’ research self-efficacy.

### 5.4. The Indirect Associations of AI Technology Readiness via AI Trust

The influence of AI technology readiness facilitators on research self-efficacy was significantly and positively mediated through AI trust. This result corroborates Horowitz and Kahn (2024) on the importance of trust as a determinant of whether people follow AI recommendations. Similarly to the significance of teacher trust as a prerequisite for adopting AI in teaching (Viberg et al., 2025), master’s student trust in AI emerges as a key factor that allows the continued use of AI tools in daily research practices in AI-embedded research contexts. AI trust can indeed reduce technology anxiety and facilitate technology continuance (Zhou et al., 2025). Trust is also important in influencing whether people are willing to depend on, allow task delegation to, and interact with AI-based systems (Berente et al., 2021). Since trust reduces risk and knowledge uncertainty in AI-enabled research, it is expected that students with high trust are more likely to use AI tools increasingly for diverse research tasks, including literature synthesis, data analysis, and manuscript preparation. This repeated, goal-directed interaction produces accumulative mastery experiences, as identified by Bandura (1997) to be the most powerful source for self-efficacy, which in turn directly enhances students’ perceptions of their ability to perform independent research. In this regard, AI trust acts as a cognitive filter through which technology readiness translates into research-related behavioral engagement, which is further linked with higher research self-efficacy. Yet, this affirmative pathway implies a properly calibrated and balanced degree of trust in AI; too much trust might have negative effects at least on the research-related cognitive processes (Zhai et al., 2024). As the current research captured perceived research self-efficacy, not actual AI-use patterns, dependency or objectively measurable research success, higher confidence in and intention to use the AI does not necessarily translate to better or more adaptive research engagement (Lau et al., 2026).

### 5.5. Multigroup Analysis Results

The multi-group analysis showed that disciplinary background did not significantly moderate the proposed relationships. This indicates that the effects of AI technology readiness, AI trust, supervisor academic support, and research self-efficacy are generally similar. This is true for both master’s students from two different disciplinary clusters. One cluster consists of the “humanities and social science” group. The other is science, engineering, and medicine. This uniformity aligns with meta-analytic evidence. The relationships defined in the Technology Readiness Index have applicability across a range of different technologies and contexts of use (Blut & Wang, 2020; A. Parasuraman & Colby, 2015). This homogeneity is also in line with Social Cognitive Theory. The theory suggests that self-efficacy beliefs are informed by similar types of informational sources across content domains (Bandura, 1997). From a practical perspective, the observed structural equivalence has more applications when designing initiatives. Uniform interventions to increase AI technology readiness, AI trust, and supervisor academic support may be developed through this uniform design.

## 6. Theoretical and Practical Implications

### 6.1. Theoretical Implications

First, whereas previous technology readiness research has predominantly focused on consumer behaviors in e-commerce contexts or K-12 educational settings, this study offers a preliminary extension of the Technology Readiness Index (TRI) model to the contexts of higher education and graduate academic practices. The findings provide tentative insights into how AI technology readiness, AI trust, and supervisor academic support are associated with research self-efficacy in AI-assisted graduate research training.

Second, supervisor support is a key environmental factor perceived by graduate students. The findings suggest that supervisor academic support is positively associated with research self-efficacy. AI technology readiness facilitators are also positively associated with perceived supervisor academic support. The indirect associations between readiness facilitators and research self-efficacy via supervisor academic support further highlight the potential interplay between personal and environmental factors. These associations become particularly relevant as AI becomes embedded in graduate academic practices. The results are consistent with the applicability of Social Cognitive Theory in contemporary higher education contexts. They also offer preliminary evidence from the graduate student population. This evidence is suggestive of the potential relevance of Social Cognitive Theory in AI-integrated learning environments.

Finally, the multigroup analysis compared the humanities and social sciences group with the science, engineering, and medicine group. The results showed no statistically significant between-group differences in path coefficients. These findings offer preliminary evidence that the associations among AI technology readiness, AI trust, supervisor academic support, and research self-efficacy may be structurally similar across disciplinary contexts.

### 6.2. Practical Implications

Three tentative suggestions are offered for supervisors of master’s students. First, given the observed association between supervisor academic support and research self-efficacy, supervisors may consider sustaining and deepening process-oriented academic guidance. This guidance includes timely feedback, methodological advice, and hands-on support. Such guidance may represent a potentially important element in AI-assisted research training. Second, given the positive association between AI technology readiness facilitators and perceived supervisor support, supervisors may wish to cultivate students’ technology optimism and innovativeness. They can do this by demonstrating concrete, discipline-relevant examples. These examples should show how AI tools may contribute to specific research tasks. Examples include literature synthesis, data analysis, and manuscript refinement. Third, in light of the observed positive association between readiness inhibitors and AI trust, supervisors may consider fostering students’ calibrated, critical trust in AI systems. They should not seek to eliminate skepticism entirely. Structured workshops could integrate AI literacy with critical-thinking development. In principle, such workshops could equip students with the evaluative competencies necessary to engage with AI as a methodologically rigorous research partner. Students should not adopt AI tools uncritically. The effectiveness of such workshops remains to be examined in future intervention studies. As the structural associations were statistically equivalent across the humanities and social sciences group and the science, engineering, and medicine group represented in this sample, these tentative suggestions may apply uniformly within these two disciplinary groupings.

Several tentative directions may also be considered for higher education policymakers. First, AI literacy cultivation systems could be developed for graduate students across disciplines. These systems could combine application-oriented training with explicit cultivation of critical evaluation capabilities. Application-oriented training may include AI-assisted text mining, data analysis, and model simulation. Second, support and training for supervisors’ educational capabilities in the intelligent era could be strengthened. Special funds and training programs might be established. These programs could enhance supervisors’ academic guidance competencies in AI-enabled graduate research. These responsibilities could be explicitly incorporated into performance evaluation and incentive systems. Interdisciplinary seminars could also be organized. These seminars could share effective supervisory practices. Finally, a safe and clearly defined environment for AI-assisted research innovation could be established. Policy frameworks might create institutionally sanctioned spaces for experimental AI use. These spaces should be accompanied by transparent ethical guidelines. These guidelines would govern AI-assisted academic research at the graduate level. They would clarify boundaries regarding academic integrity, transparency, and accountability. This would alleviate concerns that may discourage faculty and students from exploring AI technologies. We emphasize that these directions are tentative recommendations. Their effectiveness should be examined in future intervention and evaluation studies.

## 7. Limitations and Directions for Future Research

This study provides new insights into how AI technology influences graduate academic practices across disciplines; it nevertheless has several limitations. First, as for the sampling, the participants were all drawn from a Chinese geographical and cultural context. This may cause a systematic sampling bias and reduce the cross-cultural generalizability of the results. Furthermore, the inclusion criterion limited the sample to master’s students who demonstrated extensive usage of AI. This may introduce a selection bias. Second, the disciplinary classification in this study is binary. The humanities and social sciences were grouped into one category. Science, engineering, and medicine were combined into another. However, this classification does not account for possible differences in knowledge production models or technology use within these similar disciplinary groupings. Third, the cross-sectional design does not allow for causal inference. The cross-sectional methodology also constrained the study’s ability to capture how research self-efficacy and its predictors evolve longitudinally. Fourth, the model does not account for some potentially relevant covariates. These include previous research experience of students, year of study, academic performance, general digital literacy and AI-use frequency. Fifth, there remains a conceptual proximity between supervisor academic support and research self-efficacy. This overlap may have inflated the observed association between supervisor academic support and research self-efficacy.

Given these constraints, further research could be extended in the following directions. Firstly, it might be possible to extend the sample to other countries, regions or cultures and to make comparisons. Master’s students with different degrees of AI use should also be included. This would enable testing the generalizability of the current study for non-regular AI users. Second, more differentiated disciplinary divisions, based on a larger and more even number of subgroups, could be introduced. Such designs may examine the behavior, and possibly also research self-efficacy, of graduate students from comparable or related fields of study as they engage with AI in their studies. Third, longitudinal designs and/or mixed-methods approaches could be considered by researchers in the future. Data on tracking, interviews, or observations could also be added. They would more fully and dynamically show the correlations of the variables. Fourth, the researchers may also consider controlling for potential covariates statistically. These include previous research experience, year of study, academic performance, general digital literacy, and use of AI. This would allow for a more precise isolation of the net effects among the focal constructs.

## 8. Conclusions

This study examined how AI technology readiness, AI trust, and supervisor academic support are associated with the research self-efficacy of master’s students. It also tested whether these associations differ across disciplinary groupings. Cross-sectional data were collected from 425 master’s students at 19 Chinese universities. All participants self-reported regular use of AI tools in their research activities. The data were analyzed using PLS-SEM with multigroup analysis.

Three main findings emerged. First, AI technology readiness facilitators were positively associated with AI trust and with perceived supervisor academic support, whereas their direct association with research self-efficacy was not statistically significant. Second, supervisor academic support was significantly associated with research self-efficacy. Significant indirect associations were also observed between readiness facilitators and research self-efficacy via supervisor academic support and via AI trust. Readiness inhibitors showed a positive association with AI trust and a significant indirect association with research self-efficacy via AI trust. Third, partial measurement invariance was established across disciplinary groups, and no between-group differences in path coefficients reached statistical significance. The proposed model therefore appears to operate in a structurally equivalent way across the humanities and social sciences grouping and the science, engineering, and medicine grouping.

This study makes two contributions. Theoretically, it extends the Technology Readiness Index from technology adoption outcomes to efficacy beliefs in research training contexts. The results also indicate that readiness is linked to research self-efficacy primarily through supervisor academic support and AI trust, rather than through a direct path. Practically, the findings inform discipline-uniform strategies for AI literacy education and supervisory support among master’s students who regularly use AI for research purposes.

## Figures and Tables

**Table 1:** Descriptive Statistics of the Sample.

Characteristic	*n*	%
Gender		
Male	145	34.10%
Female	280	65.90%
Place of Origin		
Urban	215	50.60%
Rural	210	49.40%
Discipline		
Law	43	10.10%
Management	25	5.90%
Education	61	14.40%
Economics	11	2.60%
History	4	0.90%
Literature	15	3.50%
Arts	18	4.20%
Philosophy	4	0.90%
Science	65	15.30%
Engineering	158	37.30%
Medicine	21	4.90%
Master’s Students	425	100.0%
Using AI to Assist Research and Learning	425	100.0%

**Table 2:** *R*^2^ and *Q*^2^.

Constructs	*R* ^2^	*Q* ^2^
S	0.257	0.242
TR	0.586	0.581
RSE	0.486	0.294

**Table 3:** VIF Test Results.

Path	VIF
TR → RSE	2.424
TRIA → TR	1.487
TRIA → S	1.487
TRIA → RSE	2.777
TRIB → TR	1.487
TRIB → S	1.487
TRIB → RSE	1.552
S → RSE	1.352

**Table 4:** Hypothesis Testing Results.

Path	*β*	*t*	*p*	95% Confidence Interval		Result	*f* ^2^
				2.5%	97.5%		
**Direct Effects**							
TRIA → RSE	0.117	1.415	0.157	−0.041	0.288	Not Supported	0.010
TRIB → RSE	0.019	0.365	0.715	−0.084	0.126	Not Supported	0.000
TRIA → S	0.460	6.029	0.000	0.311	0.606	Supported	0.192
TRIB → S	0.075	1.072	0.284	−0.059	0.214	Not Supported	0.005
S → RSE	0.436	6.016	0.000	0.288	0.570	Supported	0.274
TRIA → TR	0.665	15.365	0.000	0.573	0.743	Supported	0.718
TRIB → TR	0.157	3.486	0.000	0.075	0.252	Supported	0.192
TR → RSE	0.266	3.781	0.000	0.121	0.395	Supported	0.057
**Indirect Effects**							
TRIA → S → RSE	0.201	4.478	0.000	0.118	0.290	Supported	-
TRIB → S→ RSE	0.033	1.036	0.300	−0.025	0.101	Not Supported	-
TRIA → TR → RSE	0.177	3.695	0.000	0.080	0.269	Supported	-
TRIB → TR → RSE	0.042	2.493	0.013	0.014	0.079	Supported	-

**Table 5:** Results of Measurement Invariance Testing Using Permutation (MICOM).

Constructs	Configurational Invariance (Step 1)	Compositional Invariance (Step 2)		Partial Measurement Invariance	Equal Mean Assessment (Step 3a)			Equal Variance Assessment (Step 3b)			Full Measurement Invariance
		Original Correlation	5.0%		Original Differences	95% Confidence Interval	Equal	Original Differences	95% Confidence Interval	Equal	
TR	Yes	1.000	0.999	Yes	0.151	[−0.188, 0.189]	Yes	0.242	[−0.291, 0.278]	Yes	Yes
TRIA	Yes	1.000	1.000	Yes	0.160	[−0.196, 0.191]	Yes	0.145	[−0.341, 0.333]	Yes	Yes
TRIB	Yes	1.000	0.997	Yes	0.061	[−0.192, 0.189]	Yes	0.349	[−0.262, 0.256]	No	No
S	Yes	1.000	1.000	Yes	0.029	[−0.186, 0.189]	Yes	0.435	[−0.370, 0.378]	No	No
RSE	Yes	1.000	1.000	Yes	0.122	[−0.191, 0.196]	Yes	0.243	[−0.287, 0.300]	Yes	Yes

**Table 6:** Results of Multigroup Analysis.

Path	Science/Engineering/Medicine Sample			Humanities/Social Sciences Sample			Path Coefficient Differences	*p* Value Henseler’s MGA	*p* Value Permutation Test	Decision
	Path Coefficient	CI (95%)	*p*	Path Coefficient	CI (95%)	*p*				
**Direct Effects**										
TRIA → RSE	0.014	[−0.195, 0.256]	0.901	0.241	[0.042, 0.444]	0.020	−0.227	0.145	0.186	Not Supported
TRIB → RSE	−0.004	[−0.144, 0.145]	0.960	0.065	[−0.058, 0.195]	0.317	−0.069	0.484	0.544	Not Supported
TRIA → S	0.395	[0.186, 0.591]	0.000	0.601	[0.453, 0.730]	0.000	−0.206	0.099	0.197	Not Supported
TRIB → S	0.107	[−0.076, 0.302]	0.269	−0.003	[−0.111, 0.102]	0.961	0.110	0.329	0.489	Not Supported
S → RSE	0.463	[0.271, 0.638]	0.000	0.383	[0.211, 0.541]	0.000	0.081	0.525	0.612	Not Supported
TRIA → TR	0.722	[0.602, 0.812]	0.000	0.550	[0.375, 0.689]	0.000	0.172	0.068	0.054	Not Supported
TRIB → TR	0.122	[0.020, 0.238]	0.028	0.237	[0.082, 0.404]	0.004	−0.115	0.252	0.222	Not Supported
TR → RSE	0.352	[0.134, 0.543]	0.001	0.167	[−0.011, 0.356]	0.075	0.186	0.185	0.193	Not Supported
**Indirect Effects**										
TRIA → S → RSE	0.183	[0.086, 0.313]	0.002	0.230	[0.133, 0.347]	0.000	−0.047	0.548	0.631	Not Supported
TRIB → S → RSE	0.050	[−0.030, 0.158]	0.292	−0.001	[−0.044, 0.041]	0.962	0.051	0.322	0.478	Not Supported
TRIA → TR → RSE	0.254	[0.102, 0.410]	0.001	0.092	[0.000, 0.215]	0.085	0.163	0.086	0.090	Not Supported
TRIB → TR → RSE	0.043	[0.008, 0.106]	0.073	0.040	[−0.000, 0.114]	0.152	0.004	0.907	0.913	Not Supported

Note. Following J. F. Hair et al. (2022), the BCa bootstrap confidence interval is adopted as the criterion for statistical significance; an effect is considered statistically significant when its 95% BCa confidence interval does not include zero.

## Data Availability

Data are available upon reasonable request.

## Abbreviations

The following abbreviations are used in this manuscript:

RSE	Research self-efficacy
TRI	AI Technology Readiness
TRIA	AI Technology Readiness Facilitators
TRIB	AI Technology Readiness Inhibitors
S	Supervisor Academic Support
TR	AI Trust
SEM	Science/Engineering/Medicine Sample
HSS	Humanities/Social Sciences Sample

## Appendix A

**Table A1:** AI Technology Readiness Scale.

Constructs		Item	
AI Technology Readiness Facilitators	Optimism	TRI1	Artificial intelligence technology significantly enhances my efficiency in research and daily life.
		TRI2	Artificial intelligence technology helps me manage daily learning and research tasks more efficiently in mobile scenarios.
		TRI3	Using AI tools enables me to balance academic work and personal life more flexibly.
		TRI4	Artificial intelligence technology enhances my ability to acquire cutting-edge information in my professional field.
	Innovativeness	TRI5	I often recommend AI tools suitable for research scenarios to my classmates.
		TRI6	In my social circle, I am usually among the early adopters to try new AI technologies.
		TRI7	I am capable of independently exploring and mastering AI technology tools relevant to my major.
		TRI8	I regularly pay attention to the latest developments in artificial intelligence technology within my research field.
AI Technology Readiness Inhibitors	Discomfort	TRI9	When using AI, I worry that operational errors may lead to data leakage or academic misconduct.
		TRI10	Existing AI tool tutorials are overly professional, making them difficult to apply quickly.
		TRI11	The design of some AI tools fails to adequately consider the actual needs of ordinary graduate students.
		TRI12	Many AI tools lack clear operational guidelines specifically for academic research scenarios.
	Insecurity	TRI13	Over-reliance on AI tools (for thesis polishing, data analysis, etc.) may lead to the degradation of my independent thinking abilities.
		TRI14	Over-reliance on AI technology may distract my attention and even direct me toward erroneous content.
		TRI15	AI reduces my communication with supervisors and classmates, lowering the quality of my interpersonal relationships.
		TRI16	When using AI for academic research and learning, I often feel uncertain and uneasy.

### Appendix A.1

**Table A2:** Supervisor Academic Support Scale.

Constructs		Item	
Supervisor Academic Support	Academic Task Support	S1	My supervisor establishes clear research goals for me to achieve.
		S2	My supervisor helps me plan and manage the various research tasks I need to complete.
		S3	My supervisor helps me establish task schedules and deadlines to ensure I complete tasks on time.
		S4	My supervisor provides sound and practical advice on how to plan and conduct research.
		S5	My supervisor provides advice on how to locate learning/research resources I need.
		S6	My supervisor guides me in searching for literature and research materials related to my research topic.
		S7	My supervisor seeks out information helpful for my thesis/research.
		S8	My supervisor has taught me the knowledge and skills needed to complete my research.
		S9	My supervisor spends time helping me learn the skills needed to complete my research.
		S10	My supervisor provides practical help when I need assistance.
		S11	My supervisor helps me develop good writing skills (e.g., expression of ideas, grammar, thesis structure, etc.).
	Availability and Timeliness of Feedback	S12	My supervisor sets aside uninterrupted time to meet with me regarding my research and is available to answer any of my questions at any time.
		S13	My supervisor responds to my questions or requests within a reasonable timeframe.
		S14	Whenever I submit written assignments to him/her, my supervisor provides timely feedback and answers any of my questions.

### Appendix A.2

**Table A3:** Research Self-Efficacy Scale.

Constructs	Item	
Research Self-Efficacy	RSE1	Under my supervisor’s guidance, I can conceive research topics with research significance and practical value.
	RSE2	I can proficiently use Chinese and English literature databases (e.g., CNKI, Web of Science) to retrieve literature on specific research topics.
	RSE3	Under my supervisor’s guidance, I can systematically organize and write literature reviews on specific research topics.
	RSE4	Under my supervisor’s guidance, I can formulate a research question with scientific value.
	RSE5	Under my supervisor’s guidance, I can select appropriate research methods for my research questions (e.g., experimental methods, questionnaire surveys, interviews).
	RSE6	Under my supervisor’s guidance, I can write research proposals that meet standards and pass my supervisor’s review.
	RSE7	I can systematically collect and organize research data (e.g., experimental operations, questionnaire surveys, transcription of in-depth interviews, or text coding).
	RSE8	Under my supervisor’s guidance, I can use appropriate data management and analysis tools to process research data.
	RSE9	Under my supervisor’s guidance, I can complete data analysis and identify the most meaningful findings.
	RSE10	Under my supervisor’s guidance, I can reasonably interpret data analysis results and avoid overgeneralization.
	RSE11	Under my supervisor’s guidance, I can extract the most valuable research conclusions based on theoretical and empirical analysis, and identify limitations.
	RSE12	Under my supervisor’s guidance, I can write dissertations or journal manuscripts that meet academic standards.
	RSE13	I can effectively present research findings in group meetings or academic conferences.
	RSE14	I can comply with academic ethical norms (e.g., citation formats, experimental ethics approval).
	RSE15	I can comply with graduate student academic integrity rules (e.g., avoiding plagiarism, data fabrication).

### Appendix A.3

**Table A4:** AI Trust Scale.

Constructs	Item	
AI Trust	TR1	I believe that using artificial intelligence can help me achieve better grades in my studies.
	TR2	I believe that the information provided by artificial intelligence is truthful, transparent, and reliable.
	TR3	I believe that artificial intelligence should be incorporated into the graduate education system.
	TR4	I believe that the government should increase policy support for the application of artificial intelligence in research and learning.
	TR5	I believe that using artificial intelligence in my learning is safe and reliable.

**Table A5:** Construct reliability and convergent validity of the measurement model.

Constructs	Item	Outer Loading	Cronbach’s Alpha	CR	AVE
AI Technology Readiness Facilitators	TRI1	0.902	0.965	0.965	0.774
	TRI2	0.916			
	TRI3	0.914			
	TRI4	0.924			
	TRI5	0.907			
	TRI6	0.830			
	TRI7	0.908			
	TRI8	0.875			
AI Technology Readiness Inhibitors	TRI9	0.742	0.933	0.934	0.639
	TRI10	0.857			
	TRI11	0.878			
	TRI12	0.856			
	TRI13	0.817			
	TRI14	0.844			
	TRI15	0.790			
	TRI16	0.806			
Supervisor Academic Support	S1	0.928	0.990	0.990	0.876
	S2	0.926			
	S3	0.848			
	S4	0.959			
	S5	0.964			
	S6	0.952			
	S7	0.934			
	S8	0.955			
	S9	0.949			
	S10	0.960			
	S11	0.958			
	S12	0.953			
	S13	0.927			
	S14	0.949			
Research Self-Efficacy	RSE1	0.885	0.982	0.982	0.811
	RSE2	0.761			
	RSE3	0.912			
	RSE4	0.927			
	RSE5	0.945			
	RSE6	0.928			
	RSE7	0.909			
	RSE8	0.934			
	RSE9	0.941			
	RSE10	0.939			
	RSE11	0.940			
	RSE12	0.909			
	RSE13	0.868			
AI Trust	TR1	0.883	0.927	0.927	0.717
	TR2	0.837			
	TR3	0.907			
	TR4	0.902			
	TR5	0.868			

**Table A6:** Heterotrait–Monotrait ratio (HTMT).

Constructs	TR	TRIA	TRIB	S	RSE
TR					
TRIA	0.792				
TRIB	0.566	0.588			
S	0.439	0.514	0.340		
RSE	0.575	0.565	0.384	0.621	
